# Dynamic Mitotic Localization of the Centrosomal Kinases CDK1, Plk, AurK, and Nek2 in *Dictyostelium amoebae*

**DOI:** 10.3390/cells13181513

**Published:** 2024-09-10

**Authors:** Stefan Krüger, Nathalie Pfaff, Ralph Gräf, Irene Meyer

**Affiliations:** Department of Cell Biology, University of Potsdam, Karl-Liebknecht-Str. 24-25, 14476 Potsdam-Golm, Germany; krueger33@uni-potsdam.de (S.K.); rgraef@uni-potsdam.de (R.G.)

**Keywords:** kinase, mitosis, centrosome, *Dictyostelium*, CDK1, cyclin, Aurora, Nek2, Plk

## Abstract

The centrosome of the amoebozoan model *Dictyostelium discoideum* provides the best-established model for an acentriolar centrosome outside the *Opisthokonta*. *Dictyostelium* exhibits an unusual centrosome cycle, in which duplication is initiated only at the G2/M transition and occurs entirely during the M phase. Little is known about the role of conserved centrosomal kinases in this process. Therefore, we have generated knock-in strains for Aurora (AurK), CDK1, cyclin B, Nek2, and Plk, replacing the endogenous genes with constructs expressing the respective green fluorescent Neon fusion proteins, driven by the endogenous promoters, and studied their behavior in living cells. Our results show that CDK1 and cyclin B arrive at the centrosome first, already during G2, followed by Plk, Nek2, and AurK. Furthermore, CDK1/cyclin B and AurK were dynamically localized at kinetochores, and AurK in addition at nucleoli. The putative roles of all four kinases in centrosome duplication, mitosis, cytokinesis, and nucleolar dynamics are discussed.

## 1. Introduction

The centrosome is the largest known protein complex of the cell and functions as the main microtubule organizing center (MTOC) during interphase and as spindle poles during mitosis. It consists of about one hundred different proteins (depending on the species) forming a highly organized structure [1]. There is consensus that centrosomes were already part of the inventory of the last eukaryotic common ancestor (LECA) [2,3]. Overall, there are considerable differences in centrosome structure in different model organisms. We can distinguish between centriole-containing and acentriolar centrosomes. Centriole-containing centrosomes are found in all organisms capable of forming cilia, at least in some cell types or developmental stages (i.e., animals and many others). On the other hand, acentriolar centrosomes are typically found in organisms without cilia, including many fungi and amoebozoans [2]. Acentriolar centrosomes have been intensely investigated in yeast, where they are called spindle pole bodies, and in the amoebozoan model organism *Dictyostelium discoideum* [1]. While fungi and animals are in the same eukaryotic supergroup (Opisthokonta), the *Dictyostelium* centrosome provides the best-established model for an acentriolar centrosome, outside the Opisthokonta. The *Dictyostelium* centrosome (also called nucleus-associated body—NAB) consists of a cylindrical core structure with three major layers surrounded by a corona, in which γ-tubulin-containing nodules are embedded. During interphase, it is tightly attached to the cytosolic side of the nucleus via a Sun1-containing linker [4,5]. On the nuclear side, this linker is coupled to the centromere cluster so that centrosomes and centromeres are always in close vicinity. All centromeres of the six subtelocentric chromosomes of the haploid *Dictyostelium* genome are clustered adjacent to the centrosome throughout interphase [6]. The *Dictyostelium* centrosome duplicates exactly once per cell cycle, which is also the case for centriolar centrosomes. But unlike in the latter, centrosomal duplication in *Dictyostelium* does not occur during S and G2, but during mitosis. Despite this difference, it is reasonable to assume that the duplication process involves, at least in part, conserved regulatory proteins, primarily kinases [7]. In animal cells, already in late mitosis, Polo-like kinase 1 and separase play a key role in licensing each of the two centrioles to a further round of duplication in the following cell cycle [8]. Centriole duplication is then initiated in synchrony with DNA replication through the action of cyclin-dependent kinase 2 (CDK2) [9,10,11,12]. The assembly of procentrioles at the side walls of mother and daughter centrioles requires active Polo-like kinase 4 (Plk4) [13]. In late G2, Plk1 and Aurora B kinase promote the growth of the pericentrosomal matrix and thus increase microtubule nucleation. At this time, mother and daughter centrioles, each equipped with an almost mature procentriole, are still interconnected by fibers involving rootletin and C-Nap1/Cep215. Their phosphorylation by the NIMA-related kinase Nek2 causes the disassembly of the interconnecting fibers and allows the two centrosomal entities to move apart and form the two opposing spindle poles [14,15].

In *Dictyostelium*, centrosome duplication starts at the G2/M transition [16]. First, the whole centrosome increases in size and the corona dissociates, along with the microtubule-nucleation complexes. This is accompanied by the disassembly of all preexisting microtubules. Next, the remaining core structure enters the nuclear envelope, and the central layer disappears. In prometaphase, the remaining outer layers start to separate, while each resides in its own fenestra of the nuclear envelope. At the same time, the nuclear envelope becomes highly permeable through a partial disassembly of nuclear pore complexes, which allows the exchange of spindle assembly factors and tubulin dimers during semi-closed mitosis [17,18]. The former outer layers act as mitotic centrosomes, and upon their separation, they nucleate spindle microtubules, forming a central spindle. In metaphase, astral microtubules appear. Starting with anaphase, the plaque-shaped mitotic centrosomes undergo a folding process in which the inner, microtubule-nucleating surface becomes more and more exposed to the cytoplasm. In telophase, the folding process of each mitotic centrosome completes with a scission at the kink of the fold and the re-appearance of the central layer. The new centrosomes then exit their fenestrae in the nuclear envelope but remain attached to the cytosolic surface of the nucleus. At this time the microtubule nucleating surface of the new core structure differentiates into the new corona.

Through the last decades, we have made substantial progress in characterizing the structural protein components of the *Dictyostelium* centrosome; however, our knowledge of how the dynamic process of centrosome duplication is regulated is still sparse [1]. In analogy to animal cells, kinases localizing to the centrosome in the course of its duplication are the obvious candidates to investigate. Three kinases, the only polo-like kinase Plk, the only aurora kinase AurK, and the NIMA-related kinase Nek2, have been localized to duplicating *Dictyostelium* centrosomes [19,20,21]. Yet, in all cases, their localization was studied in cells overexpressing the GFP-tagged proteins alongside the endogenous kinases. Prior to this study. Their dynamic behavior at centrosomes at natural expression levels was unknown. In addition to these kinases, cyclin-dependent kinases could play a role in centrosome duplication as well. Among the seven CDKs in *Dictyostelium discoideum* [22], CDK1 is the best candidate for a role in centrosome biogenesis, as it is active at the time of centrosome splitting.

Of the others, only Nek2 and AurK have been studied in more detail. GFP-Nek2 was assigned to the layered core structure and resided at the centrosome throughout the cell cycle [19]. The fact that overexpression of GFP-Nek2 resulted in centrosome amplification suggested a role of this kinase in the centrosome duplication process, in analogy to the splitting of duplicated animal centrosomes into two centrosomal entities at the G2/M transition.

AurK displays characteristics of both Aurora A and B kinases. As such, it is present at the centrosome only during mitosis and disappears from there during cytokinesis. It also localizes at centromeres during metaphase, at the central spindle during anaphase, and at the cleavage furrow at the end of cytokinesis [20].

In this study, we have generated knock-in strains for AurK, CDK1, cyclin B, Nek2, and Plk expressing the respective proteins via the endogenous promoter as green fluorescent Neon fusion proteins in place of the endogenous proteins and for the first time observed their behavior in live cells. Our results suggest a revision of several previous assumptions and implicate a role of these kinases in centrosome duplication, mitosis, cytokinesis, and nucleolar dynamics.

## 2. Materials and Methods

### 2.1. Cloning

Neon and tdNeon constructs: To create the C-terminal Neon knock-in constructs for CDK1, cyclin B, Plk, Nek2, and AurK, the plasmid pIS1272 was used [18]. The constructs encode a codon-optimized mNeonGreen flanked by a myc-Tag as well as a Blasticidin S resistance cassette (Supplementary Figure S1). For homologous recombination, each construct contains two homologous fragments of the targeted gene: the last 500–700 bp of the coding region without the stop codon upstream of the mNeonGreen sequence, and 500–700 bp of the 3′-region downstream of the antibiotic resistance cassette. The homologous fragments were amplified by PCR using linker primers (Fragment 1 KpnI/EcoRI and fragment 2 PstI/BamHI). As a template, genomic DNA from the parent strain AX2 was used. To generate a brighter signal for Nek2 and AurK, a tandem Neon construct was built. The sequence of the codon-optimized mNeonGreen pIS1272 [18] was used. The new, second Neon copy was created via gene synthesis (Geneart, Thermo Fisher, Darmstadt, Germany). Codons were replaced with the first or second most frequent variant, depending on which codon was already present in the first copy. The two Neon copies thus differ on the DNA level, avoiding potential issues with plasmid stability in *E. coli* or *Dictyostelium*. In addition, the two Neon fragment copies were separated by a flexible GSSGSS linker. The Nek2 and AurK td-Neon constructs were generated by insertion of the second Neon copy between the homologous 3′-fragment and the already existing Neon coding sequence via digestion with EcoRI/MfeI. For transformation into mScarlet TubA cells (IS779) or NLS td-Tomato (IS896), the plasmids were linearized by KpnI/BamHI digestion. Knock-in strains were verified via PCR using genomic DNA as a template.

### 2.2. Cell Culture

HL5c medium (Formedium, Hunstanton, UK) was supplemented with sterile filtered glucose after autoclaving. Clones were selected after the addition of 4 μg/mL Blasticidin S. For microscopy, cells were grown in adherent culture using tissue culture flasks. For Actinomycin D (AM-D) treatment, cells were allowed to settle for one hour prior to the addition of AM-D (stock solution (20 mg/mL in DMSO) at a final concentration of 100 µg/mL. The treatment was stopped by fixation. Control cells were treated with DMSO at the same concentration.

### 2.3. Live Cell Imaging

Live cell imaging was performed using a ZEISS Cell Observer SD confocal microscope (Carl Zeiss Microscopy GmbH, Jena, Germany) equipped with the Yokogawa Spinning Disk Unit CSU-X1 and two highly sensitive Evolve EM-CCD cameras (Photometrics, Tucson, AZ, USA) as described previously [18,23]. Living cells were imaged with an LCI Plan-Neofluar 63x/1.3 Imm Korr DIC objective and excited with a 488 nm (100 mW) solid-state laser using Zeiss AxioVision Rel. 4.9.1 software. Z-stack settings were set to 10 slices per stack at a frame spacing of 0.3 μm for all Neon and tdNeon knock-in cells. Stacks were recorded every 15 s. Maximum intensity projections of image stacks were calculated using Fiji Software (ImageJ 1.54f; Java1.8.0_172) [24]. For live cell imaging, cells were allowed to settle in glass bottom dishes (FluoroDish, WPI, Berlin, Germany) for at least 30 min. The medium was replaced by a Low Fluorescence (LoFlo) medium pH 6.5 (Formedium, Hunstanton, UK), and PenStrep was added (100 U/mL Penicillin, 100 µg/mL Streptomycin). Cells were incubated for at least 17 h in the dark at 21 °C. The medium was replaced with fresh LoFlo medium supplemented with ascorbic acid at a final concentration of 2 mg/mL to reduce phototoxic effects during imaging.

### 2.4. Immunofluorescence Microscopy

For immunofluorescence microscopy of cells expressing Neon or tdNeon fusion proteins, we fixed cells for 5 min with 0.5% glutaraldehyde in modified PHEM buffer (12 mM PIPES, 5 mM HEPES, 1.6 mM EGTA, 1 mM MgCl_2_, pH 7 supplemented with 0.5% Triton X-100). Subsequently, excess glutaraldehyde was inactivated by treatment with 0.1% NaBH in phosphate buffer for 5 min. Cells were counterstained with anti-α-tubulin antibody YL1/2 [25] and anti-rat AlexaFluor 568 (Thermo Fisher Scientific, Darmstadt, Germany) or anti-Cenp68 antibody [26] and anti-rabbit AlexaFluor 568 (Thermo Fisher Scientific, Darmstadt, Germany). DNA was stained with Hoechst 33342. Image acquisition and microscopy were performed at a Zeiss AxioObserver system equipped with a Zeiss Axiocam 506 mono, a PlanApo 1.4/100× lens, and ZEN 2012 Software (blue edition), including the iterative deconvolution module (Carl Zeiss Mikroskopie GmbH, Jena, Germany).

### 2.5. Statistical Analysis

For statistical analysis of CDK1 and cyclin B localization, five different cultures from one stock in six-well plates were grown. From each well, one coverslip was prepared. From each coverslip, 100 cells were analyzed. A mean value and the standard deviation for the five cultures were calculated.

## 3. Results

**Figure 1:**
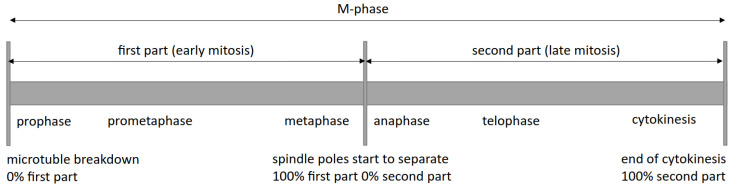
Schematic representation of the mitosis of *Dictyostelium discoideum* including cytokinesis.

Analysis of the role of regulatory proteins for centrosome duplication and mitosis first requires knowledge about the cell cycle stages at which the respective proteins localize to mitotic structures, such as spindle poles, spindle microtubules, and the midbody. In living cells, such localization studies are usually achieved by the expression of the respective proteins of interest as fluorescent fusion proteins. Based on previous studies, the kinases AurK, CDK1, Nek2, and Plk were the most interesting candidates. Regulatory proteins are expected to reside only temporally at specific structures, i.e., during their requirement for the start of the regulatory process. However, from previous experiments, we know that centrosomal proteins, which reside only temporally at centrosomes, permanently localize there when expressed at elevated levels. Therefore, we decided to generate individual knock-in strains to express each of the four kinases as green fluorescent Neon fusion proteins. In our knock-in strategy [27], due to homologous recombination of the linearized promoter-less construct, the respective endogenous gene is replaced by the C-terminally tagged construct, whose expression is then driven by the endogenous promoter. As CDK1 always requires the corresponding cyclin for activity, we also included cyclin B (CycB) in this study. We used Neon as the green fluorescent fusion partner since it exhibits the brightest fluorescence of all the fluorescent proteins we have tested in *Dictyostelium*. As a reference and to facilitate the assessment of the mitotic stage during live cell imaging, we also expressed α-tubulin as an N-terminal fusion to red fluorescent mScarlet. The latter is a suitable indicator since in *Dictyostelium* the G2/M transition and the start of centrosome duplication are marked by the breakdown of interphase microtubules, which becomes apparent by a brief disappearance of the mScarlet-α-tubulin signal from the centrosome [23]. Labeling with mScarlet-α-tubulin also allows to subdivide M phase into an early part from prophase till metaphase (first part) and a late part from anaphase till cytokinesis, starting with the time point of elongation of the short metaphase spindle. Live cell imaging of mitoses is not an easy task in *Dictyostelium* since they are more sensitive to phototoxic effects than usual cultured mammalian cell cells. Therefore, we have used a spinning disk confocal microscope and applied a couple of optimizations of imaging conditions that we have described previously [23]. As *Dictyostelium* amoebae are difficult to synchronize in mitosis, we used cultures in their logarithmic growth phase and started imaging in an arbitrarily chosen field of view. At optimal conditions, there usually occurs at least one mitosis in the field of view within a couple of hours. Since slight changes in temperature that influence the duration of the M phase cannot be avoided (times range from 8 to 14 min), we refrain here from giving exact times after which a certain event took place, as this leads to misleadingly large standard deviations when comparing different movies. Furthermore, we observed that Nek2 and Cdk1 cell lines exhibited a slightly prolonged early phase of mitosis compared to the other strains without showing otherwise any aberrant phenotype. Due to these circumstances and since our goal was not to measure exact times but to find out at which cell cycle or mitotic stage the individual kinases appeared or disappeared from the centrosome, we decided to normalize the duration of the two mitotic parts of the individual movies to 100% (Figure 1) and expressed the time points at which individual fluorescence signals appeared or disappeared as fractions of 100% (Table 1). Cultures of all knock-in strains described in this study were indistinguishable from the parent strain with regard to growth or development.

### 3.1. Nek2-tdNeon Localizes to Centrosomes Only during Early Mitosis

Among the kinases mentioned before, the NIMA-related kinase Nek2 has been studied most closely in our lab [19]. Yet, we soon realized that the expression of Nek2 via the endogenous promoter with a single Neon tag did not yield a sufficiently bright fluorescence to allow live cell imaging.

**Figure 2:**
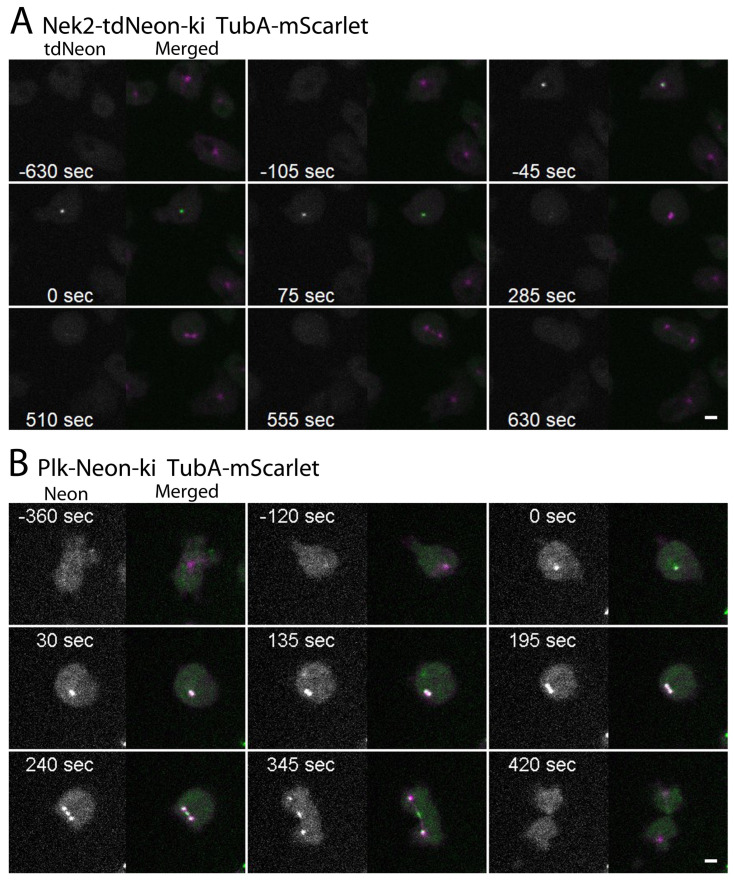
Dynamic behavior of Nek2 and Plk kinases during mitosis. Live cell spinning disk confocal microscopy of Nek2 tdNeon knock-in mScarlet-α-tubulin cells ((**A**); Video S1) and Plk Neon knock-in mScarlet-α-tubulin cells ((**B**); Video S2). Selected time points of videos S1 and S2 are shown. Time point zero is defined by microtubule breakdown. Maximum intensity projections of 11 slices per stack (z-distance 0.3 µm). Stacks were recorded every 15 s. First channel = Nek2-tdNeon/Plk-Neon (white); second, merged channel = Nek2-tdNeon/Plk-Neon (green) αTubulin-mScarlet (magenta). Bar = 3 µm.

Therefore, we created a tandem Neon tag and generated a knock-in strain expressing Nek2 with this tag, now called Nek2-tdNeon-ki. Here green fluorescent Nek2 was nicely discernible in living cells imaged with the spinning disk microscope (Figure 2A). Nek2-tdNeon started to appear above the background clearly prior to the M phase (approximately 1 min), which is indicated by the disappearance of the microtubule system (time point 0:00 s, see above and Table 1).

Nek2-tdNeon showed the strongest fluorescence during prophase and disappeared after about 50% of the first part of mitosis had passed (late prophase/early metaphase, Table 1). It showed no discernible localization other than at centrosomes/spindle poles during the period mentioned above. In contrast, when Nek2 was overexpressed as a GFP fusion protein in the context of our earlier work, it localized to centrosomes throughout the entire cell cycle [19].

### 3.2. Plk-Neon Arrives at Centrosomes Prior to Nek2 and Leaves Only during Telophase

Next, we focused on Plk, whose localization was studied earlier in a strain overexpressing a kinase-dead version of Plk as an N-terminally tagged GFP fusion [21]. Here the Plk-Neon knock-in strain with a single Neon tag was sufficiently bright to allow live cell imaging. Plk-Neon was absent from any discernible structure in interphase as well, but it started to appear at centrosomes earlier than Nek2 (approx. 2 min prior to the M phase) (Figure 2B, Table 1). It remained at mitotic spindle poles until the beginning of telophase and disappeared prior to cytokinesis. From metaphase to telophase, it was also visible in the midbody region (time point 195–345 s in Figure 2B).

### 3.3. CDK1 and Cyclin B Arrive at Centrosomes Prior to Mitosis and Leave during Anaphase

**Figure 3:**
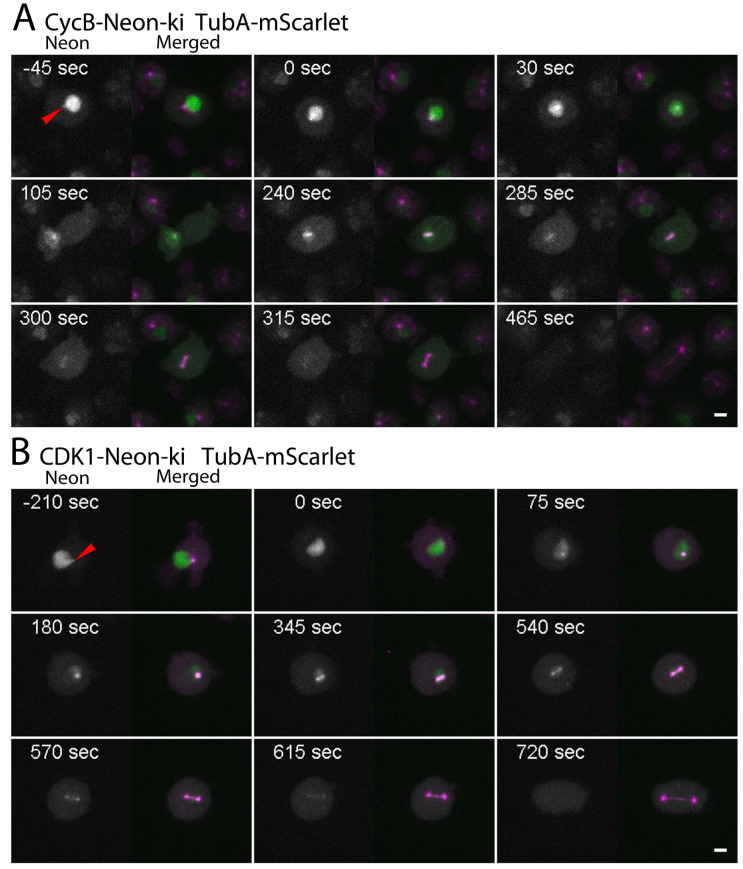
Dynamic localization of CycB and CDK1 during mitosis. Live cell spinning disk confocal microscopy of CycB Neon knock-in mScarlet-α-tubulin cells; Arrowhead shows CycB lokalisation at the centrosome in Interphase ((**A**); Video S3) and CDK1 Neon knock-in mScarlet-α-tubulin cell; Arrowhead shows CDK1 lokalisation at the centrosome in Interphase ((**B**); Video S4). Selected time points of Videos S3 and S4 are shown. Maximum intensity projections of 11 slices per stack (z-distance 0.3 µm). Stacks were recorded every 15 s. First channel = CycB/CDK1-Neon (white); second, merged channel = CycB/CDK1-Neon (green) αTubulin-mScarlet (magenta). Bar = 3 µm.

Although cyclin-dependent kinase 1 (CDK1) is considered the prototype of this whole kinase family, it has not been examined with regard to its behavior on the cellular level in *Dictyostelium*. For individual knock-in strains, CycB-Neon-ki and CDK1-Neon-ki, respectively, a single Neon tag was sufficient for live cell imaging. Prior to mitosis, i.e., in G2, CycB-Neon localizes to the nuclear interior and the centrosome (Figure 3A). The signal in the nucleus starts to disappear in prometaphase, while it remains concentrated around the centrosomes/spindle poles until anaphase (Table 1). This situation is phenocopied by CDK1-Neon (Figure 3B), whose subcellular distribution is similar to that of cyclin B with the slight but significant difference that it leaves the spindle poles slightly later than cyclin B (Table 1). The absence of both proteins from nuclei and centrosomes in telophase and many interphase cells reflects the known degradation of cyclin B by the proteasome. Although it is supposed to be stable throughout the cell cycle, CDK1 shows a similar behavior, suggesting that its presence in the nucleus and centrosomes is mediated by cyclin B. The observation that CDK1 leaves mitotic centrosomes slightly later than CycB indicates that it is associated with mitotic centrosomes also via binding to other partner proteins that lose their affinity during further mitotic progression and completion of the centrosome duplication process.

**Figure 4:**
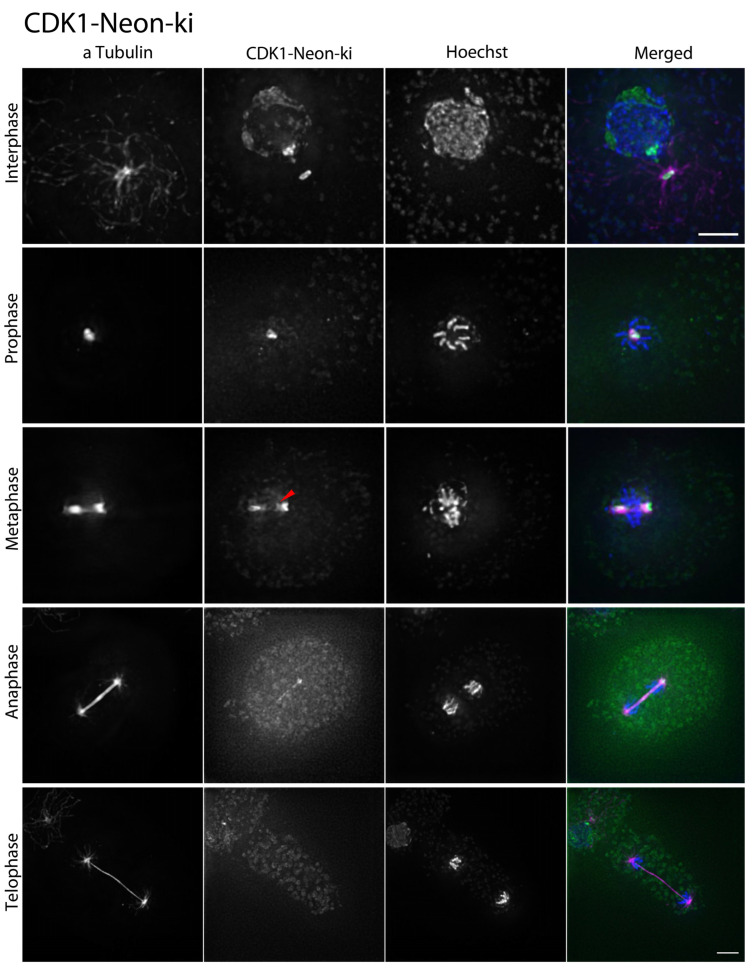
Changes of the CDK1 signal in interphase and during mitosis. Immunofluorescence microscopy of CDK1-Neon knock-in cells fixed with glutaraldehyde stained with anti-α-Tubulin and secondary antibodies anti-rat-AlexaFluor-568; Arrowhead showsa weak localization of CDK1 at centromeres remains until metaphase.Maximum intensity projections of deconvolved images (iterative DCV, measured PSF) are shown. CDK1-Neon signal in (green), α-Tubulin (red), and Hoechst (blue). Bar = 3 µm.

**Figure 5:**
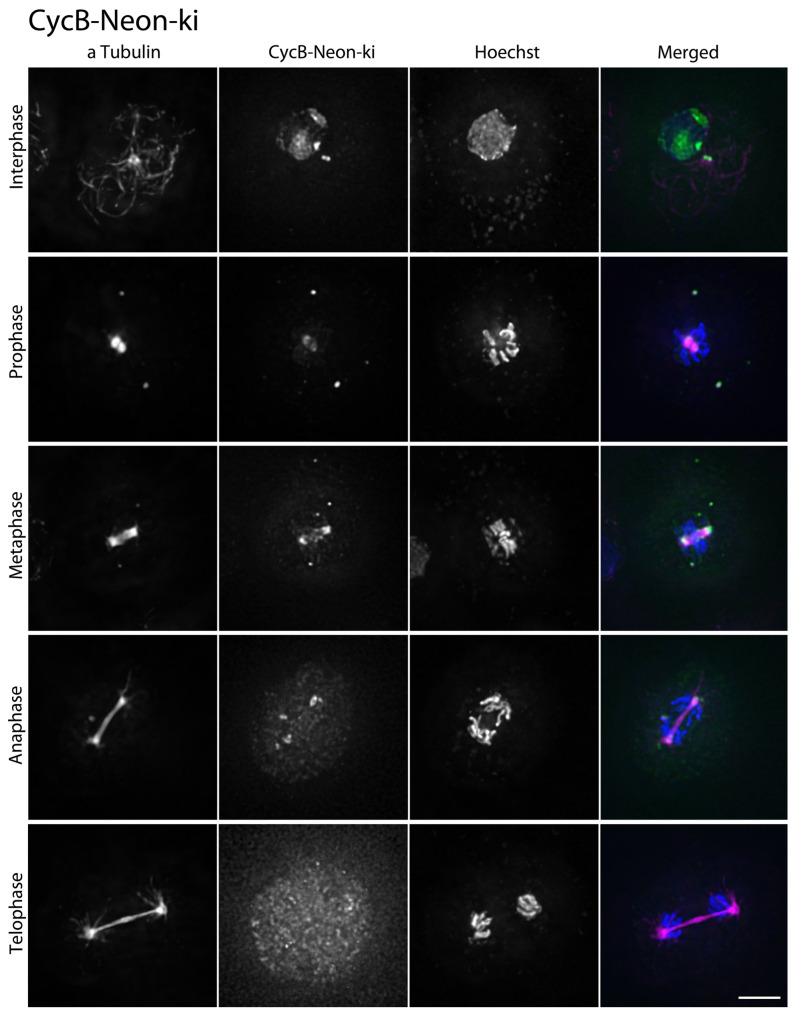
Changes of the CycB signal in interphase and during mitosis. Immunofluorescence microscopy of CycB Neon knock-in cells fixed with glutaraldehyde stained with anti-α-Tubulin and secondary antibodies anti-rat-AlexaFluor-568. Maximum intensity projections of deconvolved images (iterative DCV, measured PSF) are shown. CycB Neon signal in (green), α-Tubulin (red), and Hoechst (blue). Bar = 3 µm.

In order to assess the somewhat diffuse localization of CDK1/CycB in living cells more closely, we studied their localization also in fixed cells (Figure 4 and Figure 5). During the fixation process and permeabilization of the cells by treatment with Triton X−100, the Neon fusion proteins are washed out from localizations in living cells where they are dissolved or only loosely bound. This revealed that prior to mitosis the nuclear fraction of CDK1-Neon was found mainly at the centromere cluster (stained with the centromere marker anti-Cenp68 [26]), which is located opposite of the centrosome at the inner side of the nuclear envelope, and weakly at nucleoli, which were discernible by their intranuclear localization in the nuclear periphery, their shape, and weak staining with the Hoechst dye, which otherwise stains the whole nuclear interior in interphase cells (Supplementary Figure S2). The centrosome-bound fraction of CDK1-Neon appeared to co-localize with the outward-facing part of the centrosome since the labeled structure was darker in the center. The latter is indicative of a localization in the centrosomal corona [28]. This is in line with the observation that CDK1-Neon leaves the spindle poles prior to telophase, where a new corona is formed. It is re-acquired long after mitotic exit when the centrosomal core structure is fully covered by the new corona (see below). For sterical reasons, it is likely that proteins that are acquired later bind to the outermost structure of the centrosome, i.e., the corona. In prophase, when the centrosome has entered its fenestra in the nuclear envelope [17], the vicinity of the centrosome and the centromere cluster becomes rather close, and the two structures can no longer be resolved by conventional fluorescence microscopy. A weak localization of CDK1 at centromeres remains until metaphase (arrowhead in Figure 4).

In anaphase, we could still see a weak CDK1 signal at spindle poles and along the central spindle, before CDK1 was completely absent from all mitotic structures in telophase. CDK1 as well as cyclin B reappeared at centrosomes only ~1.5 h after the beginning of mitosis (Videos S5 and S6). This is reflected also by the absence of centrosomal CDK1 in 23 ± 2% (mean ± SD, n = 5) of unsynchronized cells.

**Figure 6:**
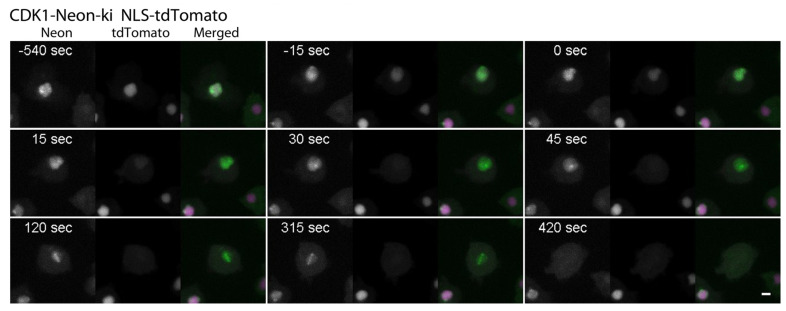
Dynamic correlation between CDK1 and NLS-tdTomato localization in the nucleus (Video S7). Live cell spinning disk confocal microscopy of CDK1-Neon ki NLS-tdTomato. Selected time points of Video S7 are shown. Time point zero is defined by nuclear envelope (NE) breakdown. Maximum intensity projections of 11 slices per stack (z-distance 0.3 µm). Stacks were recorded every 15 s. First channel = CDK1-Neon (white), second channel = NLS-tdTomato (white); third, merged channel = CDK1-Neon (green), NLS-tdTomato (magenta). Bar = 3 µm.

CycB-Neon in fixed cells was distributed in a very similar fashion as CDK1-Neon. Again, prior to mitosis, the nucleolar region and the centromere cluster were labeled within the nucleus, and the centrosome was labeled on the cytoplasmic side of the nucleus (Figure 5). Interestingly, here, CycB-Neon was typically found concentrated in two discrete dots within the microtubule-organizing center, slightly different from the CDK1-Neon distribution. In specimens prepared from unsynchronized cells, CycB-Neon was present at centrosomes in 82 ± 6% (mean ± SD, n = 5) of all cells, i.e., at a slightly higher percentage than CDK1-Neon (77 ± 2%, mean ± SD, n = 5), again suggesting that CDK1 binds to centrosomes shortly after CycB (Supplementary Figure S3). Similarly in animal cells, cyclin B is targeted to centrosomes via its hydrophobic patch and then recruits CDK1 to centrosomes [29]. The live cell observation of CDK1-Neon and CycB-Neon suggested that the disappearance of both proteins from the nuclear interior starts only after permeabilization of the nucleus in prophase, which allows entry of tubulin dimers and spindle assembly factors [17]. To investigate this more closely, we expressed our NLS-tdTomato construct in the CDK1-Neon strain. NLS-tdTomato is a tandem red fluorescent protein with a nuclear localization sequence (NLS) and is an established marker for the permeabilization of the nuclear envelope by partial disassembly of nuclear pore complexes [17,18]. Figure 6 shows clearly that the diffuse intranuclear CDK1-Neon signal starts to disappear clearly after the disappearance of NLS-tdTomato, i.e., the start of permeabilization. This suggests that CDK1 and cyclin B are not dissolved in the nuclear matrix but are associated with nuclear structures, most likely chromatin.

### 3.4. AurK-tdNeon Arrives at Centrosomes during Their Duplication and Leaves in Telophase

**Figure 7:**
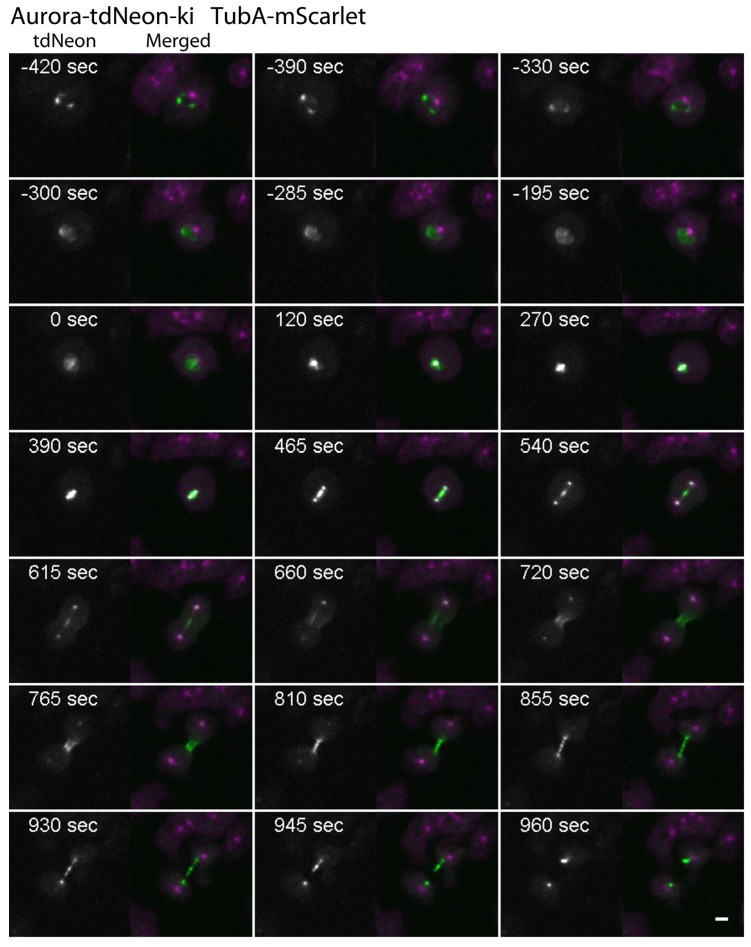
Changes in the dynamic behavior of AurK during mitosis (Video S8). Live cell spinning disk confocal microscopy of AurK tdNeon knock-in cells with mScarlet-α-tubulin. Selected time points of Video S8 are shown. Timepoint zero is defined by microtubule breakdown. Maximum intensity projections of 11 slices per stack (z-distance 0.3 µm). Stacks were recorded every 15 s. The first channel = Aurora-tdNeon (white); the second, merged channel = Aurora-tdNeon (green), αTubulin-mScarlet (magenta). Bar = 3 µm.

To study the dynamic localization of AurK, it again turned out to be necessary to use the tandem Neon tag, since the fluorescence of AurK-Neon with the single tag was too weak for live cell imaging at a sufficient quality. As for the other kinases, mScarlet-α-tubulin was expressed in the AurK-tdNeon strain. Prior to mitosis, the AurK-tdNeon signal consisted of usually two or three fluorescent spots in the nuclear periphery representing the nucleoli (Figure 7). The signal was redistributed to the centrosome at the G2/M transition prior to the disassembly of the microtubule cytoskeleton (time point 0:00), while some background in the nucleus remained.

During prophase, localization of AurK-tdNeon at the centrosome was somewhat diffuse and very similar but not identical to CDK1-Neon and CycB-Neon. While CDK1/CycB-Neon were found at the centromere cluster and the centrosome at the same time, AurK-tdNeon was found at centrosomes only a little later, at the time of centrosome splitting (7% of the first part of mitosis; Table 1). Subsequently, during prometaphase and metaphase, it became clear that AurK-tdNeon was localized to the spindle midzone in addition to the spindle poles. About 10 min after prophase, already in late telophase, the signal at the spindle midzone became weaker (time point 615 s in Figure 7), and AurK-tdNeon redistributed to two crescent-shaped regions at the constriction zone (time point 720 s in Figure 7). This appearance strongly suggested a localization at the contractile actin-myosin ring. The signal became increasingly concentrated there along with the tightening of the actin-myosin ring complex. In parallel, the signal at the spindle poles disappeared (after 72% of the second part of mitosis; Table 1, time point 810 s in Figure 7), and only a strong signal around the central spindle remained. Upon abscission of the bridge between the two daughter cells, the signal close to the abscission site remained but was distributed unevenly between both cells.

### 3.5. AurK-tdNeon Displays a Dynamic Localization at Nucleoli

**Figure 8:**
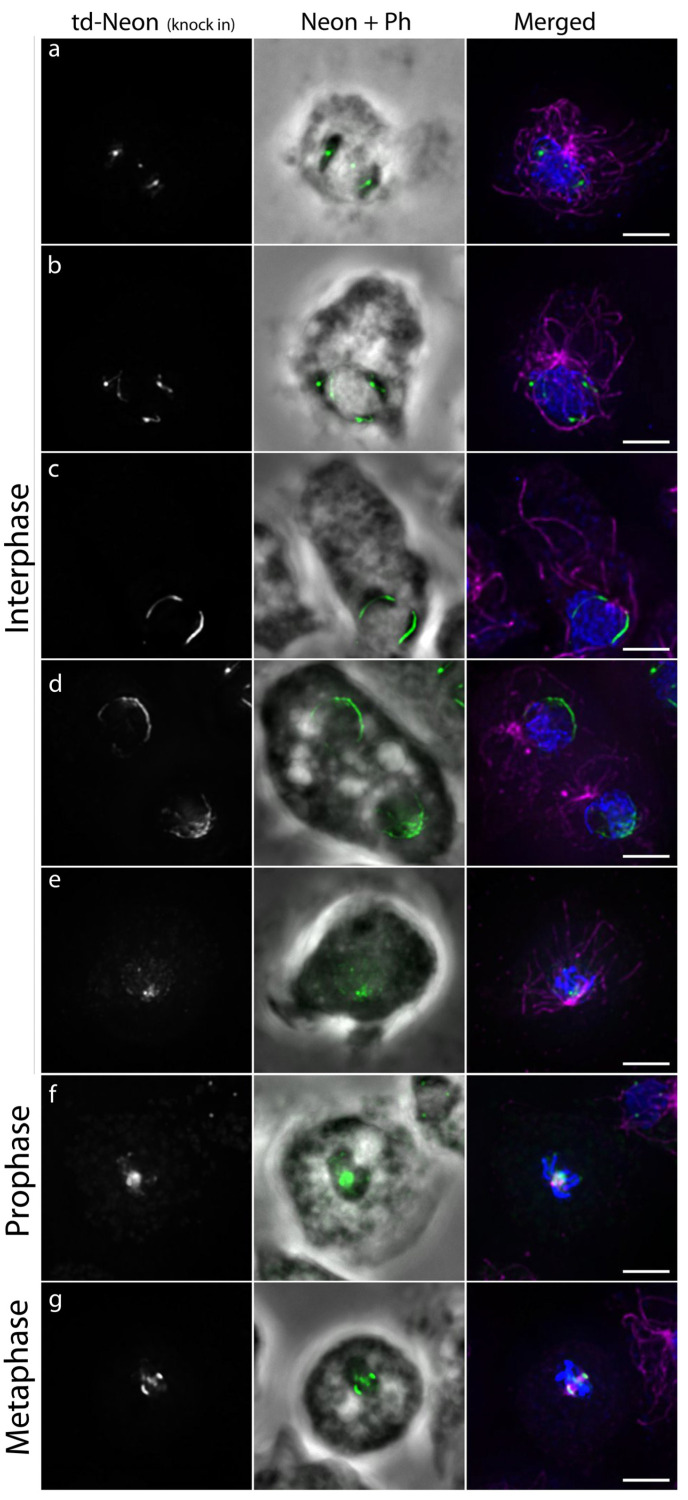
Changes of the AurK signal in the nucleus in interphase right before mitotic entry (**a**–**e**), during prophase (**f**) and metaphase (**g**). Immunofluorescence microscopy of AurK-tdNeon knock-in cells fixed with glutaraldehyde stained with anti-α-Tubulin and secondary antibodies anit-rat-AlexaFluor-568. Maximum intensity projections of the slices containing the nuclei. Overlay 1 of the Neon signal (green) with phase contrast (Ph) containing the structure of the nucleoli. Overlay 2 of the Neon signal (green) with α-Tubulin (red) and Hoechst (blue). Bar = 3 µm.

In order to elucidate the somewhat diffuse signal of AurK-tdNeon at nucleoli and during prophase more closely, we fixed AurK-tdNeon cells, co-stained them with anti-α-tubulin antibodies, and viewed cells by deconvolution fluorescence microscopy. In *Dictyostelium,* nucleoli are readily visible in phase contrast imaging as very dark regions in the nuclear periphery. AurK-tdNeon showed two different distributions at nucleoli. Either it appeared as one single spot per nucleolus or along a thin line in the periphery of the nucleolus, directly adjacent to the nuclear envelope (Figure 8a–d). During prophase (Figure 8e,f), AurK-tdNeon was present in a spotty cluster at the nuclear interior, in direct opposition to the centrosome at the outer side of the nucleus (Figure 8e). As explained above, this cluster represents the centromeres (see also Supplementary Figure S1). Centrosomal localization occurred only slightly later, at the time of centrosome splitting (Figure 8f). During metaphase, AurK-tdNeon is clearly localized at the poles and the centromeres in the equatorial region (Figure 8g).

**Figure 9:**
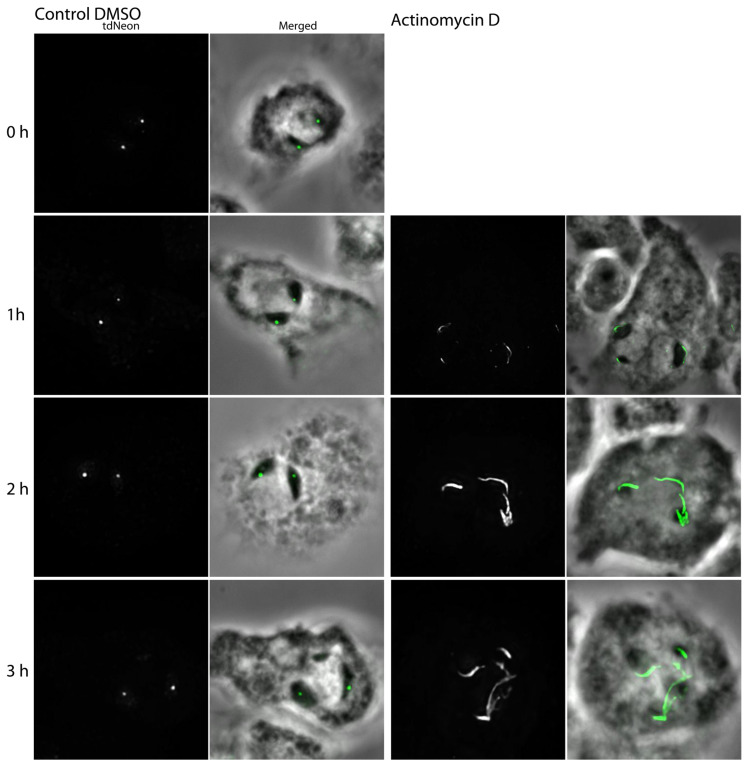
Changes of the AurK signal in the interphase nucleus upon Actinomycin-D (AM-D) treatment. Immunofluorescence microscopy of AurK-tdNeon ki cells fixed with glutaraldehyde. Maximum intensity projections are presented. Cells were treated with DMSO (control, left) or AM-D (100 µg/mL, right) and incubated for different time points (stated on the left). The left columns each show the Neon signal, while the right columns show an overlay of the Neon signal (green) with a phase contrast image, in which nucleoli appear as black structures in the nuclear periphery. Bar = 3 µm.

The differential localization of AurK-tdNeon at nucleoli was difficult to study by live cell imaging with our instrument since, at the given strength of the fluorescence signal, the high susceptibility of *Dictyostelium* amoebae to phototoxic effects [23], and the motility of the cells, the resolution was not sufficient to distinguish between the two different distributions. As nucleoli are cell cycle-dependent structures and are composed of up to four subcompartments [30], we suspected the two different subnucleolar localizations we observed for AurK-tdNeon to reflect cell cycle-dependent redistribution of the protein. The stability of nucleoli depends on RNA polymerase 1 activity, and the latter is abrogated by CDK1 phosphorylation [31]. Therefore, the transition from interphase to mitosis can also be mimicked by the treatment of cells with the RNA polymerase 1 inhibitor actinomycin D (AM-D). Most interphase cells exhibited the spot-shaped localization of AurK-tdNeon (65%), while only a few cells showed a line-shaped pattern along the nuclear envelope. About one-third of all cells showed no signal associated with nucleoli. When exponentially growing cells were treated with actinomycin D, the spot-shaped distribution found in most cells was abandoned towards the line-shaped distribution about one hour after the start of actinomycin D treatment (24% line-shaped, 25% spot-shaped, 51% without signal). In control cells treated with only the solvent DMSO, most cells (61% spot-shaped, 0% line-shaped, 39% without signal) still exhibited the spot-shaped AurK-tdNeon distribution (Figure 9).

This suggests that AurK redistributes from an association with a partner within the spot-shaped nucleolar subcompartment such as Snf12 [30] to a partner with an elongated distribution such as Src1, a LEM-family membrane protein associated with nucleoli [32].

## 4. Discussion

Here, we present the first live cell imaging study of dynamic localizations of all known *Dictyostelium* kinases relevant for the M phase from late G2 to cytokinesis, i.e., AurK, CDK1, Nek2, and Plk. For CDK1 and cyclin B in particular, prior to this work, there were no data about subcellular localization. Our study was driven by our long-standing interest in the regulation of centrosome duplication, which in *Dictyostelium* occurs entirely during the M phase.

The first steps in centrosome duplication are an increase in size and the dissociation of the microtubule-nucleating corona, which surrounds the whole layered core structure. From a mechanistic point of view, it is likely that the loss of the corona is the prerequisite for the subsequent splitting of the core structure into two centrosomal entities/spindle poles. In animal cells, the two centrosomal entities, which were formed already in the previous S phase, are connected by interconnecting fibers until late G2. At this point, C-Nap1, which is engaged in these fibers, becomes phosphorylated by Nek2 and is released from the centriole base together with the interconnecting fibers [14]. There is structural and immunological evidence that the *Dictyostelium* centrosome protein CP248 is an orthologue of C-Nap1 [1]. In analogy to the situation in animals, it is possible that phosphorylation of CP248 by Nek2 serves as the trigger for the splitting of the two centrosomal entities. Therefore, shortly after the role of Nek2 in animal cells was published [14], we identified Nek2 in *Dictyostelium* amoebae and studied its localization with the aid of an antibody and overexpression of the GFP-Nek2 fusion protein [19]. The localizations described in the latter study suggested that Nek2 is a permanent centrosomal resident, which was rather surprising for a regulatory protein. Now we show that Nek2-tdNeon is present at the centrosome only very briefly, i.e., less than a minute prior to the disassembly of the corona (indicated by microtubule breakdown) until just before centrosome splitting.

This contradiction could simply be explained by the fact that upon overexpression, Nek2 has a sufficient affinity for the centrosome even outside the G2/M transition. Permanent centrosomal localization of overexpressed proteins versus their temporal centrosomal absence at endogenous expression levels has also been known of other *Dictyostelium* proteins, e.g., CP75 [33]. With regard to our earlier immunofluorescence results, a permanent localization may also reflect a cross-reaction of anti-Nek2 with another centrosomal protein of a similar size. Anti-Nek2 was raised against bacterially expressed Nek2 and affinity purified using the antigen. In Western blots of centrosomal extracts, the antibody stained one single band at the apparent molecular weight of Nek2. Due to the parallelism of the behavior of ectopically expressed GFP-Nek2 and anti-Nek2 immunofluorescence staining, there was no reason to doubt the specificity of the antibody. Taken together, all results at that time suggested that the antibody was specific for Nek2. However, cross-reactions between different coiled-coil proteins are not unusual. For example, the similarly sized CP55 and Nek2 share such a motif. Therefore, it is possible that the permanent centrosomal residency of Nek2 revealed by both anti-Nek2 immunofluorescence microscopy and localization of overexpressed GFP-Nek2 was an artifact. The Nek2-tdNeon knock-in construct is located within the endogenous *nek2* locus, and the cells express the fusion protein driven by the endogenous promoter. Cell growth was not affected by the knock-in mutation. Therefore, we are very confident that Nek2-tdNeon shows the true localization and dynamic properties of Nek2. This is in line with the initial idea that Nek2 regulates the dissociation of the corona and would imply that it is not a structural component of the centrosome.

Permanent centrosomal residence upon overexpression was found not only for Nek2 but also for Plk [21]. Again, we show here that at endogenous expression levels, Plk-Neon was present at centrosomes only at the G2/M transition and throughout mitosis. In animal cells, Plk1 acts upstream of CDK1 by phosphorylating and activating Cdc25 phosphatase, which in turn removes inhibitory phosphate groups from CDK1 [34]. This hierarchy is most likely conserved in *Dictyostelium* since all the classical mediators of the G2/M transition are present in the *Dictyostelium* genome. However, this hierarchy of activation is not necessarily reflected by the temporal order of appearance of the respective components at centrosomes. Accordingly, in our live cell imaging studies of the Neon-tagged kinases presented in this work, CDK1 and cyclin B appeared at centrosomes already in G2 (immediately after the S phase), long before the G2/M transition when Plk arrives. This also holds true for AurK, which arrives at centrosomes even later but may still act upstream from Plk with regard to the regulation of mitotic entry. In animal cells, for example, Aurora A is on top of the activation chain since it positively regulates PLK1 already in G2 [35,36]. However, this situation is not necessarily mirrored in *Dictyostelium*, where there is only one aurora kinase, AurK, which shares properties of both Aurora A and Aurora B with regard to its localization pattern [20]. One canonical function of Aurora A is the initiation of the Ran-dependent acentrosomal spindle assembly pathway [37]. This pathway greatly increases the speed of spindle formation in animal cells by providing a large number of microtubules at the chromatin, which finally associates with kinetochores at their plus ends and can join spindle microtubules nucleated at the spindle poles [38]. This pathway, which is already initiated in G2, appears to be obsolete in *Dictyostelium* amoebae. As mentioned above, centromeres are clustered adjacent to the centrosome throughout the interphase [6]. Therefore, it is reasonable to assume that the plus ends of new microtubules nucleated at the duplicating mitotic centrosomes will meet their target kinetochores quickly enough, without the need for augmentation of spindle microtubules via a Ran or Augmin-dependent pathway (discussed in [1]). Also, it is unlikely that AurK plays a role in the initiation or the first steps of centrosome duplication, i.e., growth of the structure, dissociation of the corona, or splitting of the core structure since it appears at mitotic centrosomes only after the centrosomal core structure has separated. Therefore it is possible that AurK acts essentially as an orthologue of Aurora B in animals, i.e., it plays its first role during mitosis as a part of the chromosomal passenger protein complex (CPC) to regulate the stability of kinetochores microtubule interactions. Later on, during cytokinesis, AurK-tdNeon labeled the area of the contractile ring in the same pattern as previously published for its CPC partner protein INCENP [39]. In this respect, AurK-tdNeon labeling differs from the labeling pattern of overexpressed GFP-AurK, which at this stage unexpectedly stained only the midbody/spindle midzone and not the contractile ring and differed from the GFP-INCENP labeling pattern. Labeling of the midbody region and the uneven segregation of the labeled zone between the two daughter cells after abscission, which was also observed in this work, is in accordance with the known role of Aurora B in regulating the ESCRT complex during the abscission process [40,41].

AurK was also found at nucleoli in a differential pattern. Interestingly, this was not observed in the earlier study with GFP-AurK [20]. Yet, the localization of Aurora at nucleoli is not without precedent. For example, Aurora B was found to co-purify with human nucleoli [42]. The authors suggested that the nucleolus may function as a reservoir for a fraction of Borealin, Aurora B, and INCENP prior to mitosis. Yet, Aurora B was also found to play a direct role in nucleolar function by regulating RNA methyltransferase NSUN2 in the course of pre-mitotic nucleolus disintegration [43]. Although the nucleolus of *Dictyostelium* was not yet studied in greater detail, a set of nucleolar marker proteins was identified, and it was shown that they can be used as markers for nucleolar substructures [30,32]. In this study, nucleolar AurK-tdNeon distribution was most reminiscent of Snf12 and Src1, respectively. Labeling behavior after AM-D treatment suggested that during the cell cycle, AurK redistributes from a spot-shaped, Snf12-like localization to an elongated distribution reminiscent of Src1, which may reflect the situation prior to mitosis. However, this only reflects that AurK dynamically redistributes to different subnucleolar compartments, but it certainly requires future investigations whether these co-localizing proteins are substrates or binding partners of AurK.

A further starting point for future investigations is presented by the dynamic behavior of CDK1/cyclin B at the centrosome. Cyclin B exhibits cell-cycle-dependent degradation during mitosis and reappears after biosynthesis, while CDK1 is rather stable. The absence of clearly defined localizations of CDK1 could simply be the consequence of the absence of its targeting factor, i.e., cyclin B. The low percentage of cells without a clear localization of CDK1/cyclin B (both roughly ~20%) fits the unusual cell cycle of *Dictyostelium*, which lacks a G1 phase and has only a short S phase of 1.5 h [44]. Taking into account a usual cell cycle duration of 8–9 h in an axenic medium, this leads to the conclusion that cyclin B reappears in early G2, similar to the situation in mammalian cells. It is likely that CDK1/cyclin B is only silent resident at the centrosome prior to the removal of the inhibitory phosphates by the CDC25 phosphatase, which in turn is activated by Plk. The centrosomal phosphorylation targets of CDK1 are still unknown; however, for example, the central layer proteins CP39, CP75, and CP91 are good candidates since they all contain consensus sequences for CDK1 phosphorylation and leave the mitotic centrosomes upon centrosome splitting. Whether phosphorylation of these proteins could trigger centrosome splitting is under current investigation.

## 5. Conclusions

Expression of the kinases AurK, CDK1, cyclin B, Nek2, and Plk as neon-tagged fusion proteins as a replacement for the endogenous proteins revealed a dynamic localization of all four kinases at centrosomes. While CDK1/Cyclin B arrive at centrosomes early after completion of the S phase, Plk, Nek2, and AurK, in this order of appearance, localize to centrosomes only at or after the G2/M transition, respectively. All kinases disappear from centrosomes again during mitosis. Nek2 leaves already during metaphase, CDK1/cyclin B disappears during anaphase, while Plk and AurK dissociate from spindle poles only during telophase. In addition to centrosomes, Neon constructs labeled also the midbody region in the case of Plk and AurK. AurK furthermore displayed a dynamic localization at nucleolar substructures. CDK1/cyclin B also labeled the nuclear interior, where they associate with the centromere cluster and other sites, independently of nuclear envelope permeabilization. Taken together, we now have a solid basis to investigate the centrosomal and nuclear targets of all four kinases on a molecular basis in future studies.

## Figures and Tables

**Table 1:** Summary of live cell imaging results for CDK1, Plk, Nek2, AurK, and CycB. The time points at which the individual kinases appear or disappear at the centrosome are given as percentages of the duration of the two mitotic parts evaluated (early/late mitosis). As CycB and CDK1 appear long before the start of mitosis (approx. around the S/G2 transition; see Discussion), no values are available here. Negative values indicate a start of the event before the M phase, i.e., in G2.

Kinase	N	Signal Appears at the Centrosome—% of First Part of Cell Division	σ %	Signal Disappears at the Centrosome—% of First Part of Cell Division	σ %	Signal Disappears at the Centrosome—% of Second Part of Cell Division	σ %
CycB	4	-	-	-	-	10	6
CDK1	3	-	-	-	-	21	7
Plk	5	−68	17	-	-	97	20
Nek2	3	−18	3	50	6	-	-
AurK	5	7	2	-	-	72	30

## Data Availability

Genomic data on the genes are available at https://dictybase.org accessed on 9 July 2024. Further original data are available upon request to the corresponding author.

## Supplementary Materials

The following supporting information can be downloaded at: https://www.mdpi.com/article/10.3390/cells13181513/s1; Figure S1: Schematic of the knock-in strategy; Figure S2: Co-localization of CDK1-Neon and the centromere marker Cenp68 in prophase; Figure S3: Comparison of the percentage of cells showing mNeon signals of CDK1-Neon-Ki and CycB-Neon-ki cells at centrosomes; Table S1: list of primers used to generate genomic fragments for knock-in constructs. Video S1: Dynamic behavior of Nek2 kinase during mitosis; Video S2: Dynamic behavior of Plk kinase during mitosis; Video S3: Dynamic localization of CycB during mitosis; Video S4: Dynamic localization of CDK1 during mitosis; Video S5: Re-localization of CDK1-Neon at centrosomes during interphase; Video S6: Re-localization of CycB-Neon at centrosomes during interphase; Video S7: Dynamic correlation between CDK1 and NLS-tdTomato localization in the nucleus; Video S8: Changes of dynamic behavior of Aurora kinase during mitosis.
