# Supplementary material for: Dynamic Mitotic Localization of the Centrosomal Kinases CDK1, Plk, AurK, and Nek2 in Dictyostelium amoebae
Source: Cells. 2024 Sep 10;13(18):1513. doi: 10.3390/cells13181513 (PMC11430746; doi:10.3390/cells13181513)
Supplement: Supplementary file 1 [file cells-13-01513-s001.zip › Supplemental_data.pdf]

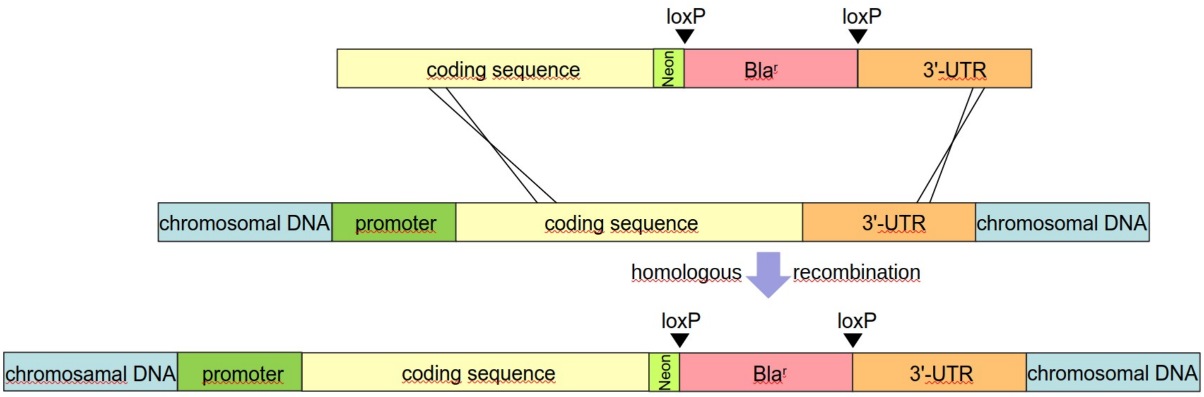

Figure S1: Schematic of the knock-in strategy used for all respective constructs. The upper bar represents the linearized DNA-construct which is transformed into Dictyostelium cells. It contains no promoter for the gene of interest, an at least 400 bp long homologous sequence for the gene of interest followed by the mNeon coding sequence, a complete blasticidin S resistance cassette, and a homologous sequence of the 3'-untranslated region (UTR) of the gene of interest. The bar in the middle represents the chromosomal DNA with the target gene. The lower bar shows the result after homologous recombination.

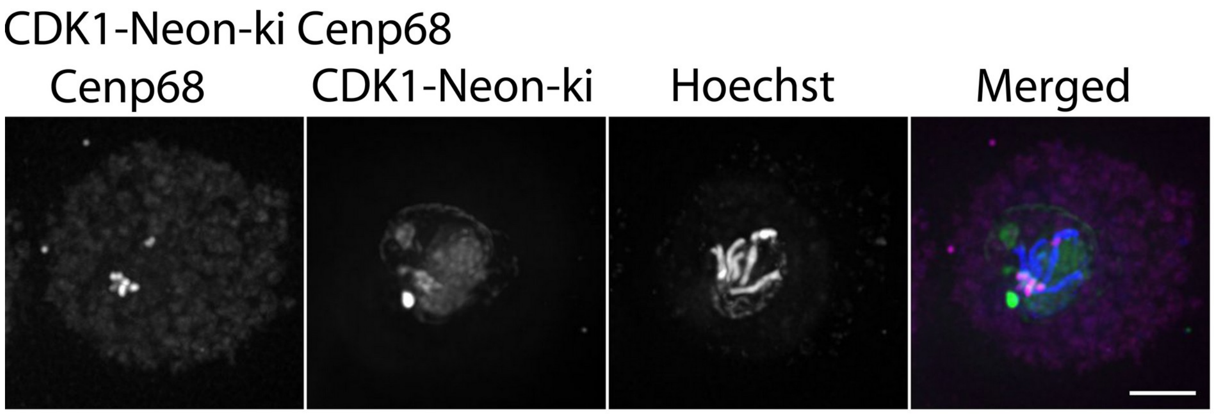

Figure S2: Co-localization of CDK1-Neon and the centromere marker Cenp68 in prophase. Immunofluorescence microscopy of CDK1-Neon knock-in cells fixed with glutaraldehyde stained with anti-Cenp68 and secondary antibodies anti-rabbit-AlexaFluor-568. Maximum intensity projections of deconvolved images (iterative DCV, measured PSF) are shown. CDK1-Neon signal in (green), Cenp68 (red) and Hoechst (blue). Bar = 3µm.;

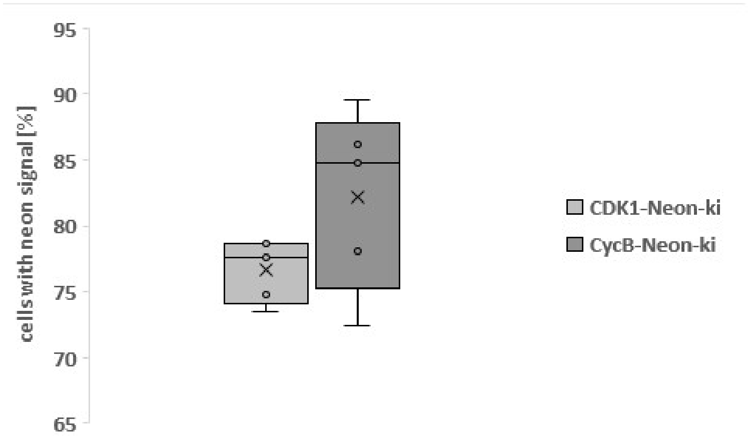

	CDK1	CycB
Mean value	77 %	82 %
standard deviation	2 %	6 %

Figure S3: Comparison of the percentage of cells showing mNeon signals of CDK1-Neon-Ki and CycB-Neon-ki cells at centrosomes. Statistical analysis of immunofluorescence microscopic images of five different cultures for each strain. For each culture a minimum of 100 cells were counted and the percentage of cells showing positive neon signals was calculated

Kinase	500-700 bp of the coding sequence	3´UTR of the gene
**CDK1**	for: aaaggtaccgcatacagtcatggtcatag<br>rev: ggggaattcgaaaaaactagtatcaagatcacc	for: ggggctgcagctaagaaatagaacttatcaacatatc<br>rev: gcatggatccaaaaacaatggttattattaaactg
**PLK**	for: aaaggtaccggtagaagagttttaaatgttactc<br>rev: ggggaattcttggttaattaattgtggttgtg	for: ggggctgcagccacaaccacaattaattaacc<br>rev: gcatggatccggaatttaattatttaccatccac
**Nek2**	for: aaaggtaccgaccaaatgtaaatgaattattagg<br>rev: ggggaattccttaaaatttgggagtggg	for: ggggctgcagcccactcccaaattttaag<br>rev: gcatggatcctcaatcgagtgtagtgtaag
**AurK**	for: aaaggtaccttttaattggtgttggtgtaag<br>rev: ggggaattcataagtcatttgagatggtaatgg	for: ggggctgcagatgtgtgattatatttgtaatatactc<br>rev: gcatggatccaattataaacctatcactaactaatgg
**CycB**	for: ctcgtcgacccacaaccaacaaatactaga<br>rev: ctcgaattcaaattcaacaactggaattgc	for: ctcctgcagaataataataataatttataatggaaattttac<br>rev: ctcggatccattatagtttattaagaaattgatctac

Table S1. List of primers used to generate genomic fragments for knock-in constructs (see also Supplementary Figure S1). Fragments had a length of 500-700bp and represented either a 3'-part of the coding sequence or the 3´untranslated region.
