# Supplementary figures and images for: Dynamic Mitotic Localization of the Centrosomal Kinases CDK1, Plk, AurK, and Nek2 in Dictyostelium amoebae
Source: Cells. 2024 Sep 10;13(18):1513. doi: 10.3390/cells13181513 (PMC11430746; doi:10.3390/cells13181513)

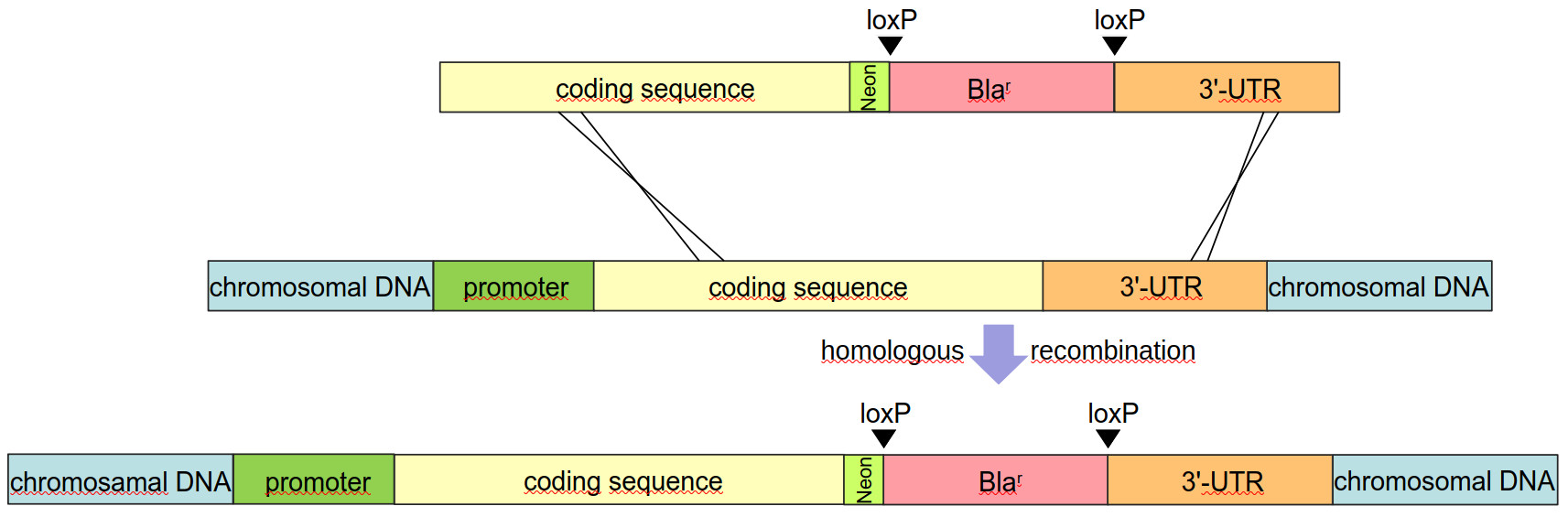

Supplement: Supplementary file 1 [file cells-13-01513-s001.zip › FigureS1.jpg]

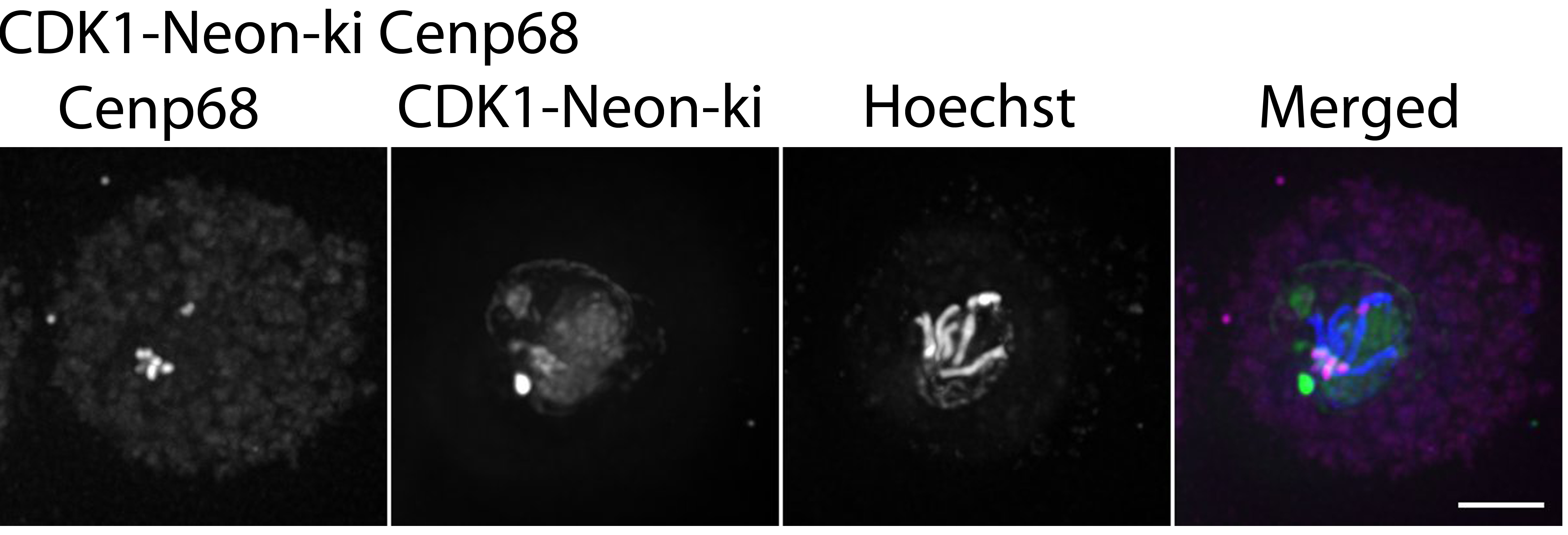

Supplement: Supplementary file 1 [file cells-13-01513-s001.zip › FigureS2.jpg]

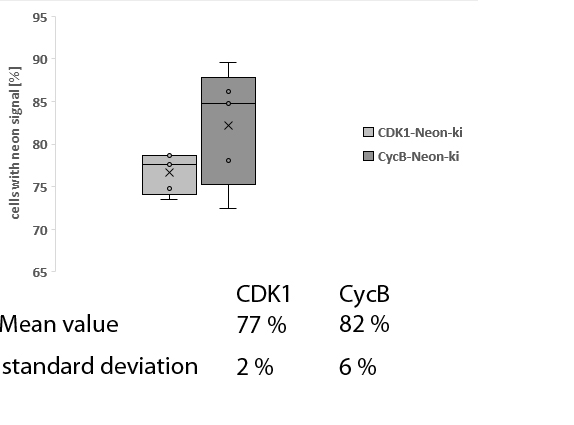

Supplement: Supplementary file 1 [file cells-13-01513-s001.zip › FigureS3.jpg]
